# Traumatic Atlanto-Axial Rotatory Fixation

**DOI:** 10.5334/jbsr.2636

**Published:** 2022-01-24

**Authors:** Damla Can, Unal Duran, Robert Ferdinand Dondelinger

**Affiliations:** 1 Centre Hospitalier Universitaire de Liège, Département de physique Médicale, Service médical de radiodiagnostic, 4000 Liège, Belgique, BE

**Keywords:** atlantoaxial fixation, AARF, torticollis, CT, trauma

## Abstract

**Teaching point:** In atlanto-axial rotatory fixation, CT in neutral position typically shows rotation of C1 on C2. Upon head rotation, atlas and axis rotate as one unit.

## Case

**Figure 1:**
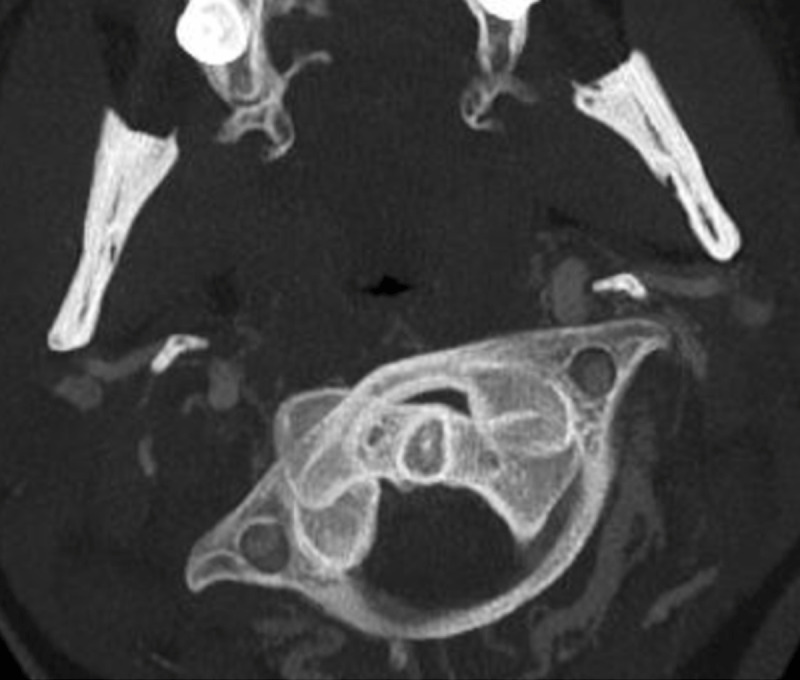
Rotation of C1 on C2 on CT in neutral position.

**Figure 2:**
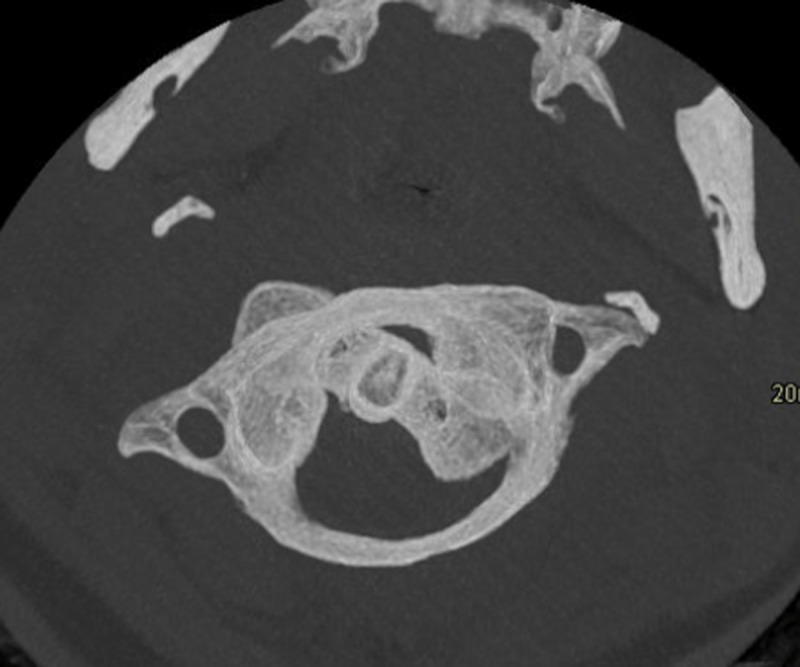
Atlas and axis rotate as one unit on CT with maximal contralateral head rotation.

A 19-year-old female presented to the emergency department for neck pain and stiffness of two weeks duration following a sharp neck movement. Axial 15-mm-thick maximum intensity projection of cervical spine CT suggested the diagnosis of Fielding type I AARF on the basis of rotation of C1 on C2 (***Figure 1***). The patient was discharged. Persistent neck pain led to re-admission. Magnetic resonance imaging (MRI) evidenced exclusive C1–C2 rotation. A ‘dynamic’ CT with maximal left-sided head rotation confirmed the fixed character of the C1–C2 complex as they rotated as a unit (***Figure 2***).

**Figure 3:**
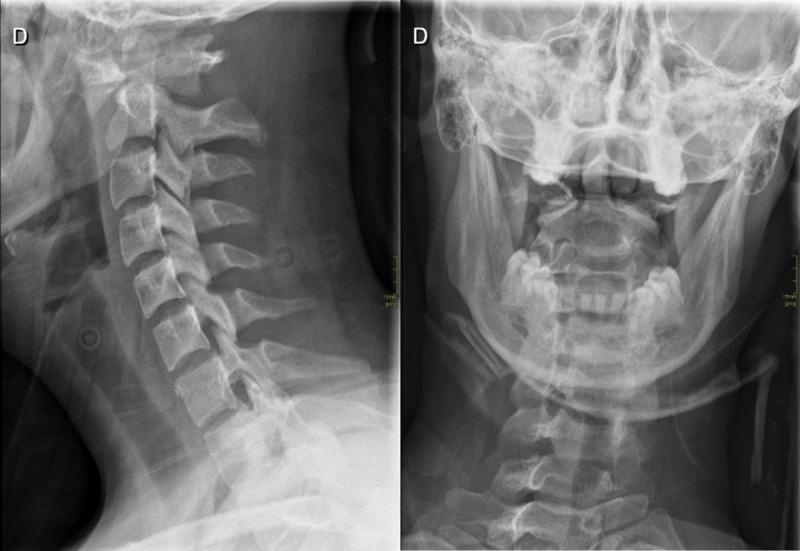
Lateral and open mouth views radiographs.

Traction anatomic repositioning preceded application of a rigid neck brace. A control radiograph demonstrated persistent rotation of C1 on C2 with one lateral mass of C1 lying anterior to the odontoid on the lateral projection and asymmetry of the lateral masses on anteroposterior open-mouth view (***Figure 3***). Repositioning was repeated with application of a halo vest, which again failed. Osteosynthesis of C1–C2 was successfully carried out.

## Comment

AARF is a rare occurrence defined by the atlanto-axial complex being maintained in a fixed position, the atlas and the axis rotating as a single unit. This entity occurs more frequently in children because of biomechanical properties of their spine. Causes include trauma, infection, or inflammation (Grisel’s syndrome), genetic conditions such as Marfan and Down syndromes, or congenital abnormalities.

Clinical symptoms like neck pain, stiffness, or torticollis are unspecific. CT is the diagnostic standard because radiographs are often difficult to interpret due to pain and deformity restricting optimum head position and the complex regional anatomy.

Fielding and Hawkins described four types of AARF on CT. Type I is the most common, without anterior displacement of the atlas and ligamentous disruption. Type II is defined by anterior C1 displacement of 3–5 mm. Type III corresponds to a rotatory subluxation with anterior displacement of more than 5 mm and type IV to a posterior displacement [1].

Dynamic CT with the head in the neutral position followed by maximal contralateral rotation of the head confirms the diagnosis of type I; the deformity remains fixed, differentiating pathology from voluntary head rotation or other causes of torticollis.

MRI provides information regarding ligamentous structures and spinal cord.

Early diagnosis of type I is imperative, as increased time interval between ‘injury’ and treatment correlates with growing failure rate of conservative management and recurrence.

Failed traction anatomic repositioning (type I), ligamentous disruption with ensuing instability (types II, III, IV), fracture, and neurologic deficit require internal fixation.

The diagnosis of AARF should be considered and prompt CT if a torticollis fails to resolve within 5–7 days, particularly if preceded by minor trauma or upper respiratory tract infection.

## References

[1] Roche CJ, et al. A pictorial review of atlanto-axial rotatory fixation: Key points for the radiologist. Clin. Radiol. 2001; 12: 947–58. DOI: 10.1053/crad.2001.0679 11795922

